# Complement component C5a Promotes Expression of IL-22 and IL-17 from Human T cells and its Implication in Age-related Macular Degeneration

**DOI:** 10.1186/1479-5876-9-111

**Published:** 2011-07-15

**Authors:** Baoying Liu, Lai Wei, Catherine Meyerle, Jingsheng Tuo, H Nida Sen, Zhiyu Li, Sagarika Chakrabarty, Elvira Agron, Chi-Chao Chan, Michael L Klein, Emily Chew, Frederick Ferris, Robert B Nussenblatt

**Affiliations:** 1 Laboratory of Immunology, National Eye Institute, National Institutes of Health, Bethesda, MD 20892, USA; 2 Division of Epidemiology and Clinical Research, National Eye Institute, National Institutes of Health, Bethesda, MD 20892, USA; 3 Macular Degeneration Center and Leonard Christensen Eye Pathology Laboratory, Casey Eye Institute, Oregon Health & Science University, Portland, OR 97239, USA

## Abstract

**Background:**

Age related macular degeneration (AMD) is the leading cause of irreversible blindness in elderly populations worldwide. Inflammation, among many factors, has been suggested to play an important role in AMD pathogenesis. Recent studies have demonstrated a strong genetic association between AMD and complement factor H (CFH), the down-regulatory factor of complement activation. Elevated levels of complement activating molecules including complement component 5a (C5a) have been found in the serum of AMD patients. Our aim is to study whether C5a can impact human T cells and its implication in AMD.

**Methods:**

Human peripheral blood mononuclear cells (PBMCs) were isolated from the blood of exudative form of AMD patients using a Ficoll gradient centrifugation protocol. Intracellular staining and enzyme-linked immunosorbent assays were used to measure protein expression. Apoptotic cells were detected by staining of cells with the annexin-V and TUNEL technology and analyzed by a FACS Caliber flow cytometer. SNP genotyping was analyzed by TaqMan genotyping assay using the Real-time PCR system 7500.

**Results:**

We show that C5a promotes interleukin (IL)-22 and IL-17 expression by human CD4^+ ^T cells. This effect is dependent on B7, IL-1β and IL-6 expression from monocytes. We have also found that C5a could protect human CD4^+ ^cells from undergoing apoptosis. Importantly, consistent with a role of C5a in promoting IL-22 and IL-17 expression, significant elevation in IL-22 and IL-17 levels was found in AMD patients as compared to non-AMD controls.

**Conclusions:**

Our results support the notion that C5a may be one of the factors contributing to the elevated serum IL-22 and IL-17 levels in AMD patients. The possible involvement of IL-22 and IL-17 in the inflammation that contributes to AMD may herald a new approach to treat AMD.

## Background

Age related macular degeneration (AMD) is clinically characterized by degenerative changes in the macula, the region of the retina that permits fine central vision. One of the key pathological features of AMD is the development of large drusen, extracellular deposits located between Bruch's membrane and the retinal pigment epithelium (RPE). These large drusen and the associated RPE changes are the major risk factors for the development of advanced AMD, which can be classified into two subtypes: dry (geographic atrophic) and wet (neovascular) [1]. Inflammation has been suggested to play an important role in AMD pathogenesis [2,3].

Genetic studies have demonstrated strong associations between AMD and several gene variants in genes coding for complement proteins, including complement factor H (CFH), factor B/C2, and C3 [4-12]. CFH is a factor that down-regulates complement activation. It is commonly thought that CFH polymorphism leads to dysregulation of alternative complement activation which may contributes to AMD pathogenesis [13]. However, the mechanism by which CFH regulates AMD progress is still not clear. Systemic activation of the complement cascade has been implicated in AMD patients [14-16]. C5a, among many alternative complement activation molecules, are elevated in peripheral blood of AMD patients [15,16]. Locally, C5a and C3a accumulate in drusen and are shown to promote choroidal neovascularization (CNV) [17], which is the hallmark of wet AMD.

Recently, a subset of effector helper T cells, IL-17-producing T cell (Th17), is implicated in the pathogenesis of various autoimmune diseases including uveitis, arthritis, multiple sclerosis, psoriasis and inflammatory bowel disease [18-20]. Proinflammatory cytokines, including IL-1β, IL-6, IL-23, IL-21 and TNFα, as well as transcription factor RORC, are responsible for differentiation and maintenance of Th17 cells within human body [21-24]. Recent evidence from the mouse suggests that C5a provides both costimulatory and survival signals to CD4^+ ^T cells and induces Th17 cytokine expression [25,26]. However, it is still not clear if C5a can impact human T cells and if Th17 cells are associated with AMD.

Here we showed that C5a protected human CD4^+ ^T cells from undergoing apoptosis and C5a promoted IL-22 and IL-17 expression from CD4^+ ^T cells of AMD patients and normal subjects as well. Intriguingly, consistent with previous observation of elevated C5a expression in the serum of AMD patients [15,16], we found significantly increased levels of IL-22 and IL-17 in the sera of AMD patients, suggesting possible roles of IL-22 and IL-17 in the inflammation that contributes to AMD.

## Methods

### Patients

PBMCs were obtained from the peripheral blood of AMD patients and healthy subjects in compliance with institutional review board (IRB) protocols after informed consent at the National Institutes of Health (NIH). The written consents were obtained. Our study has obtained ethics approval from the neuroscience IRB of NIH. AMD subjects were diagnosed with wet AMD without accompanied systemic autoimmune diseases or other immune-related diseases, as well as polypoidal vasculopathy by experienced clinicians. We excluded patients with a history of cancer within the past 5 years or patients with active inflammatory diseases. Clinical characteristics, demographic data, and single-nucleotide polymorphism information of complement associated molecules are provided in Table 1 and 2.

**Table 1:** Clinical information of AMD patients

PT number	Race, Age, Gender	Type of Disease	CFH rs1061170	C2/CFB Rs933739	C3 Rs2230199	Ocular therapy+	Co-Morbidities*	Medications++
1	W, 94, F	Wet OU	TC	GG	CC	anti-VEGF, PDT	19	H
2	W, 80, F	Wet OU	TT	GG	CC	anti-VEGF, PDT	8	W, L, X
3	W, 97, F	Wet OU	TC	GG	CC	anti-VEGF, Isteroids	none	W
4	W, 92, F	Wet OU	TC	GG	CC	anti-VEGF	14, 16, 21	Z, G, O, BB
5	W, 91, M	Wet OU	TC	GG	CC	anti-VEGF	2, 7, 18, 14	W, H, T, G, D, C, B
6	W, 91, M	Wet OU	TC	GG	CG	anti-VEGF	10, 11, 14, 15	G, C, H, B, E
7	W, 83, F	Wet OU	CC	GG	CG	anti-VEGF, PDT	8, 14, 16	W, Z, G
8	W, 79, F	Wet OD, Dry OS	CC	GG	CG	anti-VEGF, Laser Rx	14, 15, 21	W, C, G, M, BB
9	W, 74, M	Wet OU	CC	GG	GG	anti-VEGF, Laser Rx	7, 11, 14, 15	W, BB, E, Y, D, H, L, C, S
10	W, 77, M	Wet OD, Dry OS	TC	GG	CG	anti-VEGF, Isteroids	14, 15	W, L, C, H, R
11	W, 74, M	Dry OD, Wet OS	TT	GG	CC	anti-VEGF	3, 15, 16	W, Z, A
12	W, 82, F	Wet OU	TT	GG	GG	anti-VEGF, PDT	10, 11	W, E, BB, L, F
13	W, 81, F	Wet OU	CC	GG	CG	anti-VEGF, laser Rx	7, 14, 15	C, G, V
14	W, 75, M	Wet OD, Dry OS	TC	GG	CC	None	14, 15, 23	C, G, H
15	W, 67, M	Wet OD, Dry OS	TC	GG	CC	anti-VEGF, PDT	8, 15, 20	W, L, X, C, H, F
16	W, 72, F	Wet OU	TC	GG	CC	anti-VEGF, Isteroids	12, 16	Z, I, G, W
17	W, 74, M	Wet OU	CC	CG	GG	anti-VEGF, laser Rx, PDT	20	F, V
18	W, 77, F	Wet OU	CC	-	CC	anti-VEGF, PDT, Isteroid	8	X
19	W, 75, M	Dry OD, Wet OS	CC	GG	CC	anti-VEGF	11, 15	W, E, C, L
20	W, 82, M	Wet OU	-	-	-	none	3, 5, 6, 7, 11, 19,	P, B, G, A, E, D
21	W, 72, M	Wet OU	CC	GG	CG	anti-VEGF, Isteroid	7, 14	H, G, D
22	W, 83, M	Wet OD, Dry OS	TC	GG	CC	anti-VEGF, Isteroid	14, 15	W, L, H, G, C
23	W, 83, M	Wet OD, Dry OS	TT	GG	GG	anti-VEGF, PDT	14, 15, 22	B, W, G, C,
24	W, 88, F	Dry OD, Wet OS	TC	GG	CC	anti-VEGF	19	W, H,
25	W, 70, M	Wet OD, Dry OS	TC	GG	CC	anti-VEGF	10	W
26	W, 83, F	Wet OD, Dry OS	TT	GG	CC	anti-VEGF, Isteroids	8, 13, 14, 17,	Q, G, Z, W
27	W, 80, M	Dry OD, Wet OS	CC	GG	CG	anti-VEGF	7, 8, 14, 15, 21, 25	W, L, BB, Q, G, C, M, AA, D, BB
28	W, 95, M	Dry OD, Wet OS	TT	CG	CC	anti-VEGF	1, 14, 15, 22	W, E, B, K, BB, C, G, T,
29	W, 79, F	Wet OU	CC	GG	CG	anti-VEGF, PDT	7, 21, 24	W, L, D, V, CC, N
30	W, 84, M	Wet OU	TC	GG	CC	PDT, Laser Rx	5, 8	G, H, C, X, A,
31	W, 80, M	Wet OD, Dry OS	CC	CG	CG	anti-VEGF	4, 8, 15	W, BB, L, B, E, N, D, X
32	W, 97, F	Wet OU	-	-	-	None	2	J, B
33	W, 77, M	Wet OU	TC	GG	CC	anti-VEGF, Laser Rx	11	W, L, E
34	W, 57, M	Wet OU, Wet OS	TT	GG	CG	anti-VEGF	3, 7, 14, 15, 26	A, C, D, G, BB, DD
35	W, 67, F	Wet OU	CT	GG	CG	anti-VEGF	14, 15, 16, 21	G, M, Z, BB, EE
36	W, 82, F	Wet OU	CT	CG	CG	anti-VEGF	7, 8, 14, 15, 16	C, G, K, Q, Z, BB
37	W, 84, F	Wet OS	CC	GG	CC	anti-VEGF	15, 16	CC, L, G, Z,
38	W, 83, F	Wet OU	CT	GG	CG	anti-VEGF	9, 14, 15, 28	B, C, BB
39	W, 84, F	Wet OS	TT	GG	CC	anti-VEGF	16, 27	Z, CC
40	W, 90, F	Wet OU	CC	GG	CC	anti-VEGF	27	B, G, H, O, BB, CC

Ocular therapy+

anti-VEGF- either Lucentis or Avastin intravitreal injections; Isteroids-Intravitreal steroid injections; PDT- Photodynamic

therapy; Laser Rx- focal laser ablation of choroidal neovascularization

Co-morbidities*

1. anemia; 2. atrial fibrillation; 3. benign prostatic hypertrophy; 4. cerebrovascular accident; 5. COPD; 6. coronary artery disease; 7. depression; 8. diabetes; 9. Colon Cancer; 10. Glaucoma; 11. GERD; 12. gout; 13. Hashimoto's thyroiditis; 14. hypertension; 15. hypercholesterolemia; 16. hypothyroid; 17. h/o low grade vitritis; 18. Lyme Disease; 19. myocardial infarction; 20. Ocular Hypertension; 21. osteoporosis; 22. pacemaker; 23. prostate cancer; 24. seasonal allergies 25. testicular failure; 26. Asthma; 27. Uterine cancer post hysterectomy and radiation 28: Breast cancer.

Medications++

A. Anti-Benign Prostatic Hypertrophy; B. Anti-Coagulants; C. Anti-Cholesterol; D. Anti-Depressants; E. Anti-GERD; F. Anti-Glaucoma drops; G. Anti-Hypertensive medication; H. Aspirin; I. Colchicine; J. Digoxin; K. Ferrous Fumarate; L. Fish Oil; M. Fosamax; N. Gapapentin; O. Glucosamine; P. Inhalers; Q. Insulin; R. Loratadine; S. Magnesium Citrate; T. Meclizine; U. Megestrol acetate; V. NSAIDs; W. Ocuvites; X. Oral Hypoglycemic agents; Y. Scopolamine; Z. Synthroid; AA. Testosterone injections; BB. Vitamins; CC. Premarin; DD. Singulair; EE. Calcitonin.

**Table 2:** Healthy Donor Information

Donor number	Race, Age, Gender	CFH rs1061170	C2/CFB Rs9332739	C3 Rs2230199
1	W, 61, F	CT	CG	CC
2	W, 72, M	CT	GG	CC
3	W, 69, M	-	GG	GG
4	W, 71, F	CT	GG	CG
5	W, 75, M	TT	GG	CC
6	W, 65, F	TT	GG	CC
7	W, 66, F	TT	GG	CC
8	W, 66, M	CT	GG	CC
9	W, 73, M	TT	GG	CC
10	W, 61, M	CC	GG	CC
11	W, 69, M	TT	GG	CG
12	W, 73, F	CT	GG	CC
13	W, 65, F	CT	GG	CC
14	W, 75, F	CT	GG	CC
15	W, 69, M	CT	GG	CC
16	W, 65, F	CC	GG	GG
17	W, 65, F	CT	GG	CC
18	W, 65, F	-	CG	CG
19	W, 62, F	TT	CG	CC
20	W, 71, M	TT	GG	CG
21	W, 72, M	CT	GG	CC
22	W, 62, M	TT	GG	CC
23	W, 71, F	CC	GG	CG
24	W, 66, F	TT	GG	CG
25	W, 65, F	CT	GG	CC
26	W, 61, F	CT	GG	CC
27	W, 63, F	CT	CG	CC
28	W, 64, F	CT	GG	CC
29	W, 68, M	CT	CG	CG
30	W, 70, M	CT	GG	-
31	W, 87, F	-	-	-
32	W, 59, M	CC	CC	CC
33	W, 64, F	-	-	CC
34	W, 61, M	TT	GG	CG
35	W, 66, M	CC	CG	CG
36	W, 65, M	CC	GG	CG
37	W, 65, F	CT	GG	GG
38	W, 60, F	CT	GG	CG
39	W, 63, F	TT	GG	CG
40	W, 66, F	CT	CC	CC
41	W, 77, F	-	-	-
42	W, 65, M	CT	GG	CC
43	W, 66, M	CT	GG	CG
44	W, 62, M	CT	GG	CC
45	W, 66, M	-	-	-

### Cell sorting

To sort CD4^+ ^T cells and monocytes, 1 × 10^7 ^PBMCs were stained with allophycocyanin-labeled CD3 (clone UCHT1, BD Biosciences), PE-labeled CD4 (clone RPA-T4, BD Biosciences), or FITC labeled CD14 (clone M5E2, BD Biosciences) for 20 minutes in 1% BSA PBS staining buffer. Cells were then washed and subsequently sorted on a FACS Aria (BD Biosciences). BD FACSDiva software was used to sort the cells.

### Cell culture and flow cytometry

PBMC cells were cultured in RPMI 1640 medium (Invitrogen, Carlsbad, CA) containing 10% fetal bovine serum (Gemini Bioproducts, West Sacramento, CA) supplemented with 2 mM glutamine and 1× antibiotics. For T cell and monocytes separation, PBMCs were cultured in the same RPMI medium described above and then stained with anti-CD3 and anti-CD4 antibodies for T cell and anti-CD14 for monocyte separation. Cells were treated with or without C5a (50 ng/ml from R&D Systems, endotoxin level <1.1 EU per 1 μg of protein) and a C5aR antagonist (2.5 ug/ml from Jerini Ophthalmic Inc, also called JPE-1375, is a hexameric linear peptidomimetic molecule that inhibits C5a binding to the human C5aR). Anti-B7.1 and B7.2 antibodies (10 μg/ml of each) or anti-IL-1β (10 μg/ml) and anti-IL-6 (10 μg/ml) neutralization antibodies were added into the cell culture in indicated experiments. Intracellular staining was performed after 5 days of C5a culture. Cells were stimulated with PMA (10 ng/ml), ionomycin (0.5 μg/ml) and Golgistop for 4 hours at 37°C before intracellular staining. 5 × 10^5 ^cells were stained with FITC labeled CD45RA (clone HI100, BD Biosciences), PE-IL-22 (clone 22URTI, eBioscience), or PE-IL-17A (clone eBio64DEC17, eBioscience), perCP-CD4 (clone SK3, BD Biosciences) and allophycocyanin-labeled CD3. The intracellular staining procedure was based on the BD Bioscience protocol. Briefly, cells were firstly stained with cell surface markers (anti-CD4, anti-CD45RA), and then permeabilized and proceeded to intracellular staining (anti-LI-17A or IL-22). Cells were acquired by a FACSCalibur flow cytometer (BD Biosciences) and analyzed by FlowJo software (TreeStar, San Jose, CA).

### Cytokine Analysis

Sera from patients or supernatants collected from cell culture were tested by ELISA for IL-22 and IL-17, or sent for multiplex cytokine analysis (Aushon Biosystems). IL-22 and IL-17A ELISA kits were purchased from R&D Systems, Inc. (Minneapolis, MN) and were performed based on kit protocols.

### Apoptosis Assay

Apoptotic cells were detected by staining cells with both the annexin-V-FITC (BD Biosciences) and TUNEL technology (Roche, Indianapolis, IN) according to the manufacturer's instructions. Phospho-Bad expression was detected by western blot using anti-Phospho-Bad antibody (Cell Signaling Technology).

### SDS-PAGE and Western blotting

A total of 5 million T cells were lysed in 100 μl lysis buffer [50 mM Tris-Cl, 1% Triton X-100, 100 mM NaCl, 2 mM EDTA, 50 mM NaF, 50 mM glycerol-phosphate, 1 mM NaVO_4 _and 1× protease inhibitor cocktail (Roche)]. Complete cell lysis was achieved by immediately vortexing the cells and then boiling in an equal amount of 2 × SDS protein loading buffer at 95°C for 5 minutes. Cell debris was removed by centrifugation at 12, 000 rpm for 3 min. Twenty microliter of each sample was loaded into a 12% SDS-polyacrylamide gel containing a 4% stacking gel. Immunoblotting was carried out. Primary antibodies of anti-Phospho-Bad, anti-Bad were purchased from Cell Signaling Technology (Beverly, MA). Anti-β-actin antibody was from Santa Cruz Biotechnology, Inc.(Santa Cruz, CA).

### SNP Genotyping

Genomic DNA was extracted from the peripheral blood of each individual using Promega Wizard Genomic DNA Purification kit. The samples were analyzed by TaqMan genotyping assay using the Real-time PCR system 7500 (Applied Biosystems, Foster City, CA, USA). The primers and probes for *C2/CFB *rs9332739 and *C3 *rs2230199 were from the inventory SNP assay while *CFH *rs1061170 were custom-designed from Applied Biosystems. Genotypes were determined based on the fluorescence intensities of FAM and VIC. The call rates of 3 assays were >98.5% and the call accuracies (consistency of duplicate wells of selected samples) were 100%.

### Statistical Analysis

Non-parametric methods (Wilcoxon two-sample test) were used since the expression of IL17 and IL22 does not follow a parametric distribution. To evaluate if the expression of these 2 cytokines follows a normal distribution, we visually checked the histograms as well as used the Kolmogorov-Smirnov method. For the association study between IL-22/IL-17 and some characteristics of patients (CFH, C2/CFB, C3 genotypes, gender, co-morbidities of diabetes, hypertension and hypercholesterolemia), Wilcoxon's nonparametric two-sample rank sum test was used. Age was analyzed using Pearson correlation. The software used for all the analyses was "The SAS System", version 9.2.

## Results

We listed the demographic, clinical information for both controls and AMD patients in Table 1 and Table 2. Ocular therapies, co-morbidities and complement related genetic variance were also included to AMD patients for later data analysis. These information will be used to evaluate confounding factors. All the subjects in this study are Caucasians. There are 45 controls and the age range was from 59 to 87. Fifty-three percent (53%) are females and 47% are males. There are 40 AMD patients in this study and the age range was from 57 to 97. Fifty percent (50%) are females and 50% are males.

### C5a promotes the expression of IL-22 and IL-17 from human T cells *in vitro*

**Figure 1:**
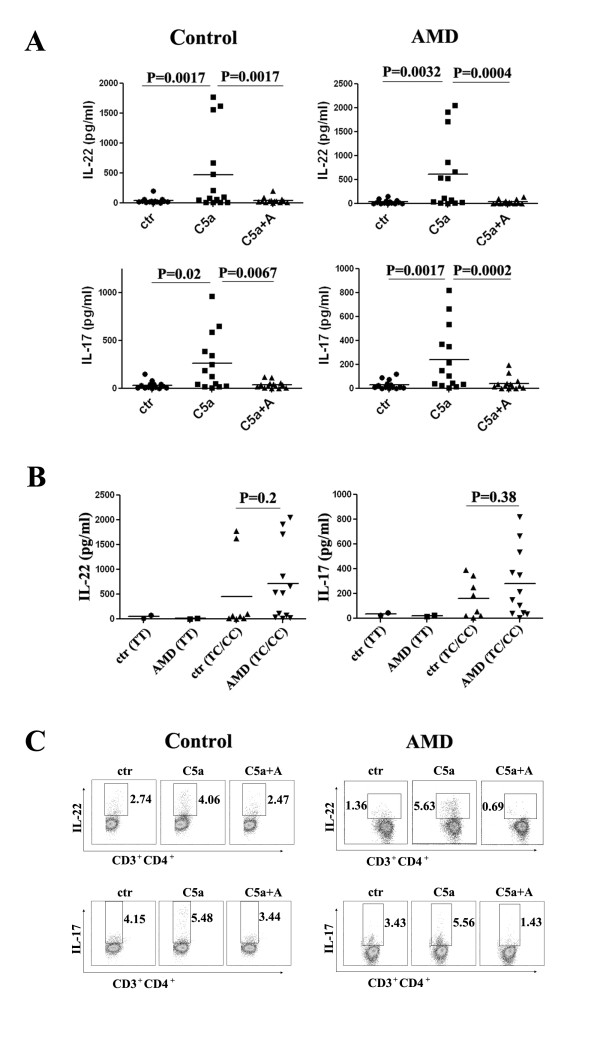
**C5a promotes the expression of IL-22 and IL-17 from T cells**. (A) IL-22 and IL-17 in 3-day culture supernatants of PBMCs from 14 AMD patients and 14 controls. (B) C5a induced IL-22/IL-17 expression in both controls and AMD patients were subgrouped based on CFH genotypes. (C) Intracytoplasmic staining of IL-22 and IL-17 from both controls and AMD patients after 5 days of culture with or without C5a and C5aR antagonist.

To study the role of C5a on human CD4^+ ^T cells, we used ELISA and intracellular staining to detect cytokine expression. PBMCs from AMD patients and controls were treated with or without C5a and a C5aR antagonist for 3 days. Cell supernatants from 14 controls and 14 AMD patients were used for ELISA analysis and are presented side by side in Figure 1A. The addition of C5a greatly increased the expression of IL-22 and IL-17A in PBMC cells from both AMD patients and controls. Blocking C5aR reversed this effect (Figure 1A). Interestingly, we cannot detect the changes of IFNκ and IL-4 levels before and after C5a treatment. We then subgrouped the C5a induced IL-22/IL-17 expression in both controls and AMD patients based on their CFH SNP information (rs1061170). As shown in Figure 1B, there was no significant difference on cytokine expression between controls and AMD patients. However, C5a high response individuals all have the risk CFH allele genotype (heterozygous/homozygous, TC/CC) in both control and patient groups. Intracellular staining data further confirmed that C5a induced IL-22 and IL-17A secretion from cultured CD3^+^CD4^+ ^T cells after PBMCs were treated for 5 days (Figure 1C).

### Monocytes are important for C5a induced IL-22 and IL-17 expression from T cells

**Figure 2:**
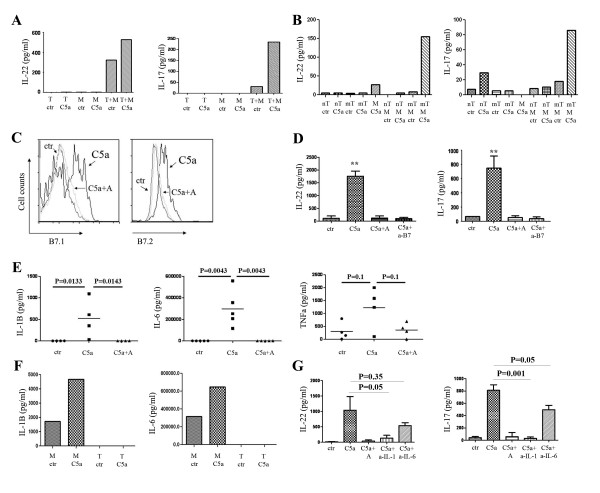
**IL-1β and IL-6 secreting monocytes are important for C5a induced IL-22 and IL-17 expression form T cells**. (A) CD3^+^CD4^+ ^T (T) cells and CD3^-^CD14^+ ^monocytes (M) were sorted and cultured with or without C5a for 3 days. Cell supernatants were assessed for IL-22 and IL-17 expression. Three separate experiments were performed and the figure shows representative data. (B) CD3^+^CD4^+^CD45RA^+ ^(naïve T cells, nT) and CD3^+^CD4^+^CD45RA^- ^(memory T cells, mT) T cells and CD3^-^CD14^+ ^monocytes (M) were sorted and cultured with or without C5a for 3 days. IL-22 and IL-17 levels were measured from supernatants. Three separate experiments were performed and the figure shows representative data. (C) C5a activates B7 expression on monocytes. PBMCs were cultured with or without C5a for 1 day. CD3^-^CD14^+ ^monocytes were gated for indicated cell markers' expression. Similar results were seen in another independent assay. (D) IL-22 and IL-17 in 3-day culture supernatants of PBMCs with the presence or absence of C5a, C5aR antagonist and anti-B7.1 and anti-B7.2 antibodies. (E) C5a stimulates monocytes to secrete IL-1β and IL-6. PBMCs were cultured with or without C5a and C5aR antagonist for 3 days. Cell supernatants were assayed for IL-1β, IL-6 and TNFα expression. (F) Monocytes and T cells were sorted and cultured with or without C5a for 3 day. Cell supernatants were assayed for IL-1β and IL-6 expression. Three separate experiments were performed and the figure shows representative data. (G) IL-22 and IL-17 in 3-day culture supernatants of PBMCs with the presence or absence of C5a with isotype control antibody, C5aR antagonist and anti-IL-1β and anti-IL-6 neutralization antibodies. Three separate experiments were performed.

To address if peripheral monocytes play a role in C5a induced IL-22 and IL-17 expression of CD4^+ ^T cells, CD14^+ ^monocytes and CD3^+^CD4^+ ^T cells were cultured separately or together, with or without C5a (50 ng/ml) for 72 hours. Protein levels of IL-22 and IL-17A in the culture supernatants were detected by ELISA. As shown in Figure 2A, IL-22 and IL-17 were barely detected in cultures with monocytes or CD4^+ ^T cells alone. Interestingly, C5a induced expression of both cytokines only in co-cultures of CD4^+ ^T cells and monocytes, suggesting that monocytes are necessary for C5a to promote IL-22 and IL-17 expression. Further experiments showed that only memory CD4^+ ^T cells, when co-cultured with monocytes, could produce Th17 cytokines (Figure 2B).

The effects of monocytes on T cells could be due to either direct interaction between B7.1/B7.2 on monocytes and CD28 on T cells, or indirect effects such as the production of cytokines. C5a treatment promoted both B7.1 and B7.2 expression on monocytes (Figure 2C). When a blocking antibody that interrupts the B7-CD28 interaction was added to the culture, the induction of both IL-22 and IL-17 by C5a was diminished, to a similar extent as the effect seen with the C5aR antagonist (Figure 2D). Previous studies have shown that IL-1β and IL-6 are drivers of Th17 cell polarization [22,27,28]. We found a significantly increased expression of both IL-1β and IL-6 in the supernatants of co-cultures containing both monocytes and T cells and an increased trend for TNF-α although P value not significant (Figure 2E), but not IFN-κ or IL-23. Both IL-1β and IL-6 were produced by monocytes (Figure 2F). We therefore neutralized IL-1β and IL-6 with neutralizing antibodies and found that the induction of IL-22 and IL-17 by C5a were significantly dampened (Figure 2G). Collectively, our results indicate that not only direct interaction between monocytes and T cells, but also the secretion of IL-1β and IL-6 by monocytes is required for promotion of Th17 cytokines by C5a.

### C5a protects T cells from undergoing apoptosis

**Figure 3:**
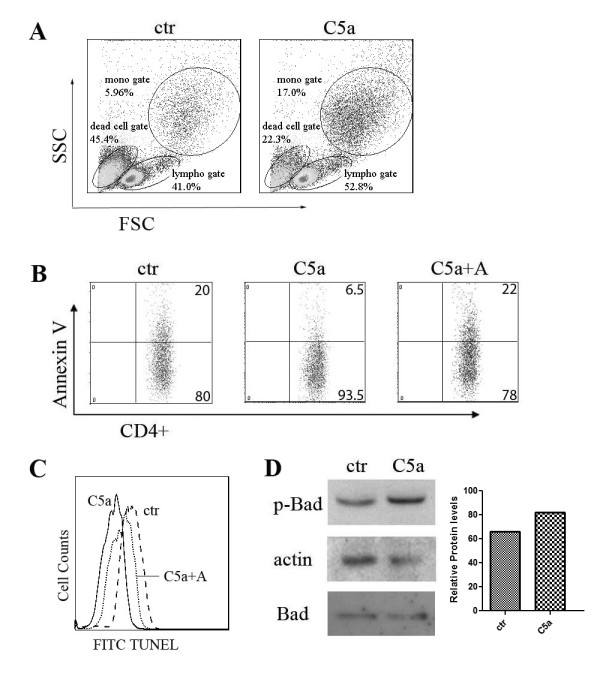
**C5a protects T cells from undergoing apoptosis**. (A) Scatter plot of PBMCs cultured with or without C5a. Three separate experiments were performed and the figure shows representative data. (B) Annexin V expression on T cells cultured with or without C5a and C5aR antagonist. Ten separate experiments were performed and the figure shows representative data. (C) TUNEL staining of CD4^+ ^T cells treated with or without C5a and C5aR antagonist. (D) PBMCs were treated with or without C5a for 2 days. T cells were sorted and processed for western blot analysis for indicated antibodies. Densitometry graph is also shown. Similar results were seen in another independent assay.

To fully understand the overall effect of C5a on CD4^+ ^T cells, we examined C5a's effect on CD4^+ ^T cell survival. Purified PBMC cells naturally undergo apoptosis in culture and they usually die without stimulation in 7 days. We added C5a with or without the C5aR antagonist to the culture for 2 days and compared the percentage of cells undergoing apoptosis for more than 10 individuals. Morphological signs of the inhibition of apoptosis, including more cell aggregates and less shrunken cells, were observed in C5a group. Figure 3A represents a typical flow cytometry scatter plot. The percentages of lymphocyte and monocyte gates increased after C5a treatment (from 41% to 52.8% and 5.96% to 17.0% respectively). Further apoptosis staining showed that the addition of C5a prevented CD4^+ ^T cells from undergoing apoptosis, as indicated by annexin V staining. This effect was abrogated by the addition of a C5aR antagonist (Figure 3B). TUNEL staining confirmed these results. Apoptotic cells were labeled with fluorescein. Fluorescein labeled cells had less intense staining in C5a treatment group as compared to the control group (Figure 3C). Moreover, the expression of Phospho-Bad, one of the anti-apoptotic indicators, was increased in CD4^+ ^T cells after C5a treatment (Figure 3D).

### Higher IL-22 and IL-17 expression in AMD patients

**Figure 4:**
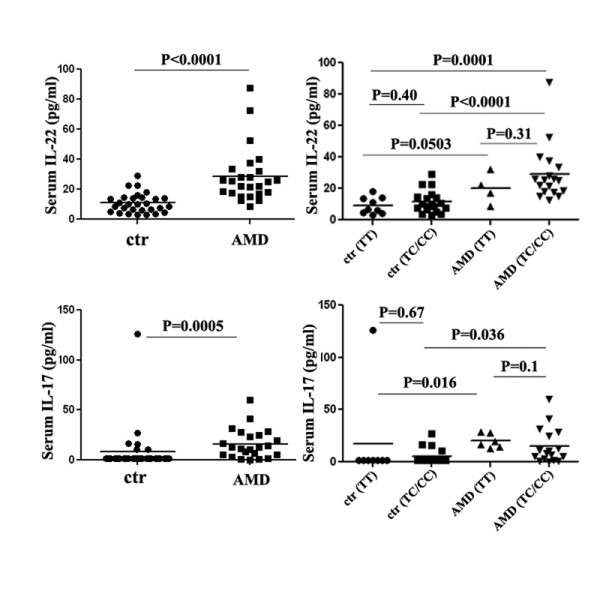
**IL-22 and IL-17 present a higher expression in AMD patients**. Sera from 29 controls and 25 AMD patients were assayed for IL-22. Thirty (30) controls and 23 AMD patients was assayed for IL-17 expression. IL-22/IL-17 expression in both controls and AMD patients were subgrouped based on the subjects' CFH genotypes.

Different cohort studies have shown elevated levels of C5a in AMD blood as compared to controls [15,16]. Based on our *in vitro *data that C5a induced Th17 cytokine expression from human T cells, we want to do a pilot study to evaluate the expression of IL-22 and IL-17 in the serum of AMD patients. As shown in Figure 4, IL-22 and IL-17 levels were significantly elevated in AMD patients compared with controls. We then subgrouped cytokine expression in both the controls and the AMD patients based on their CFH SNP information (rs1061170). As shown in Figure 4, IL-22/IL-17 cytokine high expression AMD patients have the risk CFH allele genotypes (heterozygous/homozygous, TC/CC). However, for control group, IL-22/IL-17 expressions remained low regardless of their CFH SNP genotypes. We performed the association study between IL-22/IL-17 cytokine expressions and some characteristics of patients (CFH, C2/CFB, C3 genotypes, age, gender, co-morbidities of diabetes, hypertension and hypercholesterolemia). Our results indicated that there were no statistically significant associations between IL-22/IL-17 cytokine expressions and these variances (all P values are more than 0.05, Additional file 1: Table S1).

## Discussion

In this study, we have provided evidence that C5a induced IL-22 and IL-17A expression from human CD4^+ ^T cells. Importantly, consistent with previous observations of elevated C5a expression in the serum of AMD patients from different cohorts [15,16], we observed significantly increased levels of IL-22 and IL-17A in the sera of AMD patients. However, so far, we do not have direct evidence showing that the elevated Th17 cytokine levels in AMD patients' sera are due to higher C5a expression in AMD patients. C5a may be one of the many factors related to this observed effect. Other unknown factors may also contribute to this T cell activation seen in AMD patients. Interestingly, the findings that C5a specifically promoted the Th17 family cytokine production, but not IFNκ nor IL-4, also correlated with the fact that there were similar IFNκ and IL-4 levels in the sera of AMD patients as compared to controls. The dysregulation of the complement system has been linked to multiple neurodegenerative diseases including Alzheimer's disease, Parkinson's disease, as well as AMD [29]. The induction of inflammatory Th17 cytokines, including IL-22 and IL-17, by complement component C5a could potentially elucidate the general mechanism by which inflammation contributes to the pathogenesis of these diseases previously referred to as degenerative. Our results support a role for C5a in protecting CD4^+ ^T cells from undergoing apoptosis (Figure 3). These findings suggest that the enhanced effector T cell function by C5a is at least partially mediated by limiting pro-inflammatory cell death.

We found that monoctytes are necessary for C5a induced Th17 cytokine production through two mechanisms: 1) promoting the direct interaction between monocytes and T cells; 2) indirectly stimulating the production of IL-1β and IL-6 from monocytes. C5a can bind to the trans-membrane receptors C5aR/CD88 and C5L2 (GPR77) which are expressed on monocytes. C5L2 is expressed at much lower levels as compared to CD88. C5a binding to CD88 leads to a number of functional changes including activation of inflammation. However, the pathophysiological role of C5L2 is currently controversial with both pro-inflammatory and anti-inflammatory roles reported [30]. Previous reports from rodent models have shown that C5a has a direct effect on T cells by interacting with the C5a receptor expressed on T cells, a finding which is different from what we have observed in humans [26]. Fang *et al*. recently demonstrated that C5a itself has no effect on Th17 cytokine production in mouse [31]. However, C5a synergizes with TLR4 to produce serum factors that drive Th17 cell differentiation [31]. Liu *et al*. reported that local interactions among C3a/C5a, C3aR/C5aR, antigen presenting cells (APC) and T cells are important for IFNκ and IL-17 production of T cells in a murine EAE (Experimental autoimmune encephalomyelitis) model [32]. In another murine sepsis model, Xu *et al*. shows that C5a affects the crosstalk between DC and gamma/delta T cells and results in a large production of IL-17[33]. In a human study, Hueber and colleagues showed that C5a induces IL-17 from human mast cells [34]. Our work is the first human study showing that monocytes play an essential role in C5a promoted expression of Th17 cytokines from CD4^+ ^T cells.

Several research teams have reported that a common SNP of CFH, Tyr^402^His, has a particularly strong association with AMD [4,6,8]. We sub-grouped IL-22/IL-17 expression based on the subjects' CFH SNP genotypes and found that AMD patients with higher IL-22/IL-17 cytokine expression were likely to have the risk CFH allele (TC/CC) (Figure 4). However, serum IL-22/IL-17 cytokine levels showed no difference between the two CFH genotype groups (TT versus TC/CC) in controls. These results suggest that this CFH SNP does not explain the elevated Th17 cytokine expression. However, this genetic variant may be one of the many factors influencing Th17 cytokine expression.

Dysregulation of alternative complement activation has been reported to be involved in AMD pathogenesis. The drusen of AMD donor eyes contain almost all molecules of the alternative complement pathway, including CFH, C3, C5, C3a, C5a, and the membrane attack complex (MAC) [35-37]. These results suggest the role of the complement system in the eye. The products of complement activation can also be detected in the blood of AMD patients. Scholl *et al. *[16] found higher levels of alternative complement activation molecules in the blood from an AMD cohort, including Ba, C3d, MAC, C3a, and C5a. A subsequent study in a larger independent cohort of patients and controls confirmed these results, showing the activation of the alternative pathway of complement in blood is associated with genetic polymorphisms in complement factor B and increases with age [14]. Reynolds and colleagues also found an increased plasma concentration of C5a and Bb in advanced AMD [15]. In addition, a recent report has shown that immunization with carboxyethylpyrrole generated by oxidative damage to DHA (Docosahexaenoic acid) present in the drusen and plasma from AMD-affected individuals is sufficient to produce AMD like lesions in mice and antibody titers of carboxyethylpyrrole correlates with disease pathology, suggesting the involvement of the acquired immune pathway in disease pathology [38]. In this study, we found C5a induced Th17 cytokine expression from human T cells in vitro, which correlates with the increased levels of Th17 cytokines in AMD blood. IL-22 has been shown to induce apoptosis of fetal retinal pigment epithelium (RPE) cells and reduce RPE cell electrical resistance in culture [39]. However, whether systemic observations reflects pathological events in the eye and how systemic activation may ultimately be manifest in the eye remain to be defined.

To date there is no effective treatments other than attempts to slow the progression of geographic atrophy form of AMD, while neovascular AMD is treated with anti-VEGF medications injected directly into the eye [40,41]. Previous attempts at controlling the wet form of AMD with corticosteroid therapy have shown that the beneficial effect is transient with a significant side-effect risk profile [42]. Health improving behavior (no-smoking), diet, and exercise may be preventive measures for AMD [43]. Several compounds targeting complement pathway are currently in clinical trials [13]. We recently reported that immunotherapy directed against T-cell activation resulted in patients with recurrent CNV requiring fewer injections of anti-VEGF [44]. The dysregulation of the acquired immune pathway we describe here may provide us with new therapeutic strategies.

## Conclusion

In conclusion, we have shown that C5a promoted expression of Th17 cytokines from human CD4^+ ^T cells. Consistent with several cohort observation of elevated C5a expression in the serum of AMD patients [14-16], our results support the notion that C5a may be one of the factors contributing to the elevated serum IL-22 and IL-17 levels in AMD patients. Targeting adaptive immune system, more specifically the Th17 family of cytokines, may have beneficial effect on the course of AMD.

## Abbreviations

AMD: Age related macular degeneration; CFH: complement factor H; IL: interleukin; RPE: retinal pigment epithelium; PBMC: peripheral blood mononuclear cell; SNP: single nucleotide polymorphism

## Competing interests

The authors declare that they have no competing interests.

## Authors' contributions

BL, RBN have conceived and designed the research and drafted the manuscript; BL, LW, JT, ZL, SC have performed the experiments. CM, HNS, CCC, MLK, EC, FF have provided materials and clinical samples and help analyzed the clinical data. EA, BL performed statistical analysis. All authors read and approved the final manuscript.

## Supplementary Material

Additional file 1 **Table S1 Association between the serum levels of IL-22/IL-17 with patients' characteristics**. P values were listed for the association between IL-22/IL-17 and some characteristics of patients (CFH, C2/CFB, C3 genotypes, gender, co-morbidities of diabetes, hypertension and hypercholesterolemia). Age was analyzed using Pearson correlation. Click here for file
