# Supplementary material for: Complement component C5a Promotes Expression of IL-22 and IL-17 from Human T cells and its Implication in Age-related Macular Degeneration
Source: J Transl Med. 2011 Jul 15;9:111. doi: 10.1186/1479-5876-9-111 (PMC3154861; doi:10.1186/1479-5876-9-111)
Supplement: Additional file 1 — Table S1 Association between the serum levels of IL-22/IL-17 with patients' characteristics. P values were listed for the association between IL-22/IL-17 and some characteristics of patients (CFH, C2/CFB, C3 genotypes, gender, co-morbidities of diabetes, hypertension and hypercholesterolemia). Age was analyzed using Pearson correlation. [file 1479-5876-9-111-S1.DOCX]

**Table S1 Association between the serum levels of IL-22/IL-17 with patients’ characteristics**

|  | **IL-22** | **IL-17** |
| --- | --- | --- |
|  | P value | P value |
| **Sex** | **0.10** | **0.69** |
| **CFH** | **0.31** | **0.08** |
| **C3** | **0.89** | **0.89** |
| **C2/CFB** | **0.80** | **0.10** |
| **Diabetes** | **0.55** | **0.48** |
| **Hypertension** | **0.08** | **0.56** |
| **Hypercholesterolemia** | **0.18** | **0.96** |
|  | **IL-22** | **IL-17** |
|  | Pearson correlation coefficient | Pearson correlation coefficient |
| **Age** | **0.096** | **0.072** |
